# Revealing the Therapeutic Uses of Garlic (*Allium sativum*) and Its Potential for Drug Discovery

**DOI:** 10.1155/2021/8817288

**Published:** 2021-12-30

**Authors:** Azene Tesfaye

**Affiliations:** Department of Medical Laboratory Science, College of Medicine and Health Sciences, Arba Minch University, Arba Minch, Ethiopia

## Abstract

**Background:**

Garlic is a common bulb vegetable that is used to flavor and flavor food. The plant contains biologically active components that contribute to its pharmacological properties. This paper attempts to examine the therapeutic uses and potential role in the drug development of garlic for various human diseases.

**Methods:**

To obtain crucial data and scientific knowledge about the therapeutic uses of garlic, systematic literature searches were conducted using key terms on well-known indexed platforms such as PubMed, Scopus, Web of Science, Medline, Embase, and popular search engines.

**Results:**

Garlic, which is utilized as a spice and flavoring ingredient, is found to have fundamental nutritional components. Carbohydrates, protein, fat, minerals, water, and vitamins are all found in abundance in this plant. The plant also has a high medicinal value and is used to cure a variety of human diseases. It has anti-inflammatory, rheumatological, ulcer inhibiting, anticholinergic, analgesic, antimicrobial, antistress, antidiabetes, anticancer, liver protection, anthelmintics, antioxidants, antifungal, and wound healing properties, as well as properties that help with asthma, arthritis, chronic fever, tuberculosis, runny nose, malaria, leprosy, skin discoloration, and itching, indigestion, colic, enlarged spleen, hemorrhoids, fistula, bone fracture, gout, urinary tract disease, diabetes, kidney stones, anemia, jaundice, epilepsy, cataract, and night blindness.

**Conclusions:**

The nutritional content of the plant is significant, and it has incredible therapeutic potential. The findings of this study are needed to investigate the therapeutic potential, as it may be a promising option for drug development.

## 1. Introduction

For thousands of years, humans have employed natural plant-based chemicals to cure a variety of foods [1]. Nutritional variables have an important influence on the onset of a variety of human disorders [2]. Many different diets are believed to be beneficial to human health in many cultures. Despite ethnic diversity, healthy eating habits share certain basic elements [3]. Garlic (*Allium sativum* L.) is a member of the *Alliaceae* family and, after onions, is the second most widely used *Allium*. It is widely produced globally and used as a spice, additive, and medicinal plant, as stated [4–7].

Garlic has a higher concentration of sulfur compounds (allicin, diallyl disulfide, S-allylcysteine, and diallyl trisulfide), which are responsible for its therapeutic properties. It is consumed either raw (fresh leaves or dried cloves) or processed (garlic oil, garlic extracts, and garlic powder), with varying chemical compositions and bioactive ingredient levels. It is long been known as a beneficial spice and a popular treatment for a variety of diseases and physiological conditions [8, 9].

Garlic is employed in a variety of ways as a therapeutic remedy in modern society. As a result, scientists in numerous fields are currently concentrating their efforts on determining the medicinal potential of garlic in human health [2, 10]. The broad-spectrum therapeutic effects of garlic with low toxicity are of prime interest to researchers studying its medicinal properties [2]. Garlic extract has antibacterial, fungicidal, and viral action [11]. Several authors have complimented the chemical components of garlic for the treatment of cardiovascular disease, cancer, diabetes, blood pressure, atherosclerosis, and hyperlipidemia [2, 10, 11]. As a result, this study examines the nutritional composition and medicinal uses of garlic, as well as its potential for drug development.

## 2. Methods

### 2.1. Data Sources and Data Extraction

Systematic literature searches were conducted on published articles using the key terms including allicin, ajoene, *Allium sativum*, organosulfur compounds, and *t* use therapies on a well-known index platform to acquire the needed information. The databases PubMed, Scopus, Web of Science, Medline, Embase, the Cochrane Library, WorldCat, Epistemonikos, and Science Direct, as well as the popular search engines (Pdf searcher.org, Google Scholar, Osun.org, and Science Direct), were used. Only 50 out of 200 published articles met the criteria for inclusion, and data extraction was carried out.

## 3. Result

### 3.1. Phytochemical Constituents of Garlic

**Figure 1:**
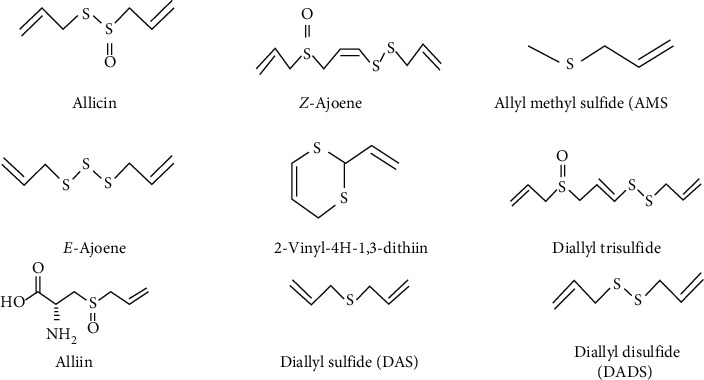
Biological active constituents and their derivatives in garlic (*Allium sativum*).

Organosulfur compounds (Figure 1) such as allicin, diallyl thiosulfonate (allicin), diallyl sulfide (DAS), diallyl disulfide (DADS), diallyl trisulfide (DATS), E/Z-ajoene [12, 13], S-allylmercapto [14–16], N-acetylcysteine (NAC) [17], and S-allyl-cysteine (SAC) are present in garlic. Among the substances identified in garlic include peptides, steroids, terpenoids, flavonoids, and saponins [18], as well as phenols. Organosulfur compounds from raw garlic are more easily digested than those from cooked garlic [18]. There are amino acids, minerals, and enzymes such as alliinase, peroxidases, and myrosinase [9], as well as selenium, germanium, tellurium, and other trace elements in addition to sulfur compounds. Garlic polysaccharides also comprise 85% fructose, 14% glucose, and 1% galactose [19].

### 3.2. Nutritional Values of Garlic

**Figure 2:**
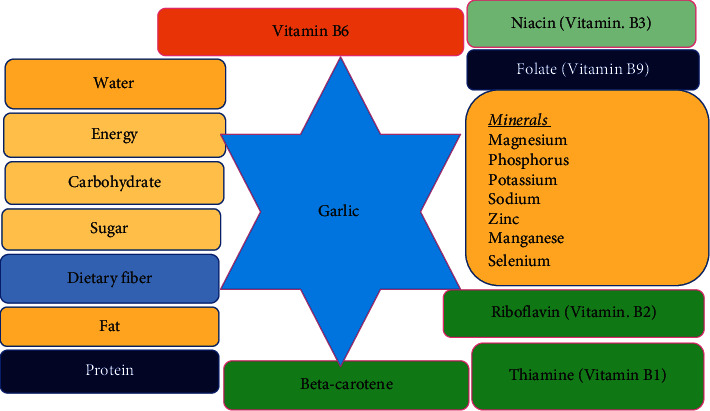
Nutritional compositions of garlic (*Allium sativum*).

Garlic is used as a spice in the food industry, both fresh and dried. It keeps track of nutritionally relevant quantities (Figure 2). In addition, sugar, protein, fat, vitamins [8, 20], calcium, potassium, phosphorus, sulfur, iodine, fiber, and silicon are all found in garlic [21]. In addition to its flavor, it contains minerals, vitamins, and other components that are good for human health [20]. Because of its pungent smell, the plant is mostly used as a condiment and seasoning for recipes that contain both green tops and bulbs. Garlic adds flavor to dishes while also making them simpler to digest. It is highly recommended in the world's most opulent kitchens.

### 3.3. Therapeutic Uses of Garlic

Because of its biologically active component, allicin, and its derivatives, garlic has long been used as a medicine to treat a variety of illnesses and disorders including high blood pressure [8], high cholesterol, coronary artery disease [22], cancers such as colon, rectal, stomach, breast, prostate, and bladder cancers, as well as lung cancer [23], and cardiovascular diseases such as antilipemic, hypotensive, enlarged prostate (BP-hyperplasia), diabetes, osteoarthritis, hay fever (allergic rhinitis), travelers' diarrhea, preeclampsia, and cold and flu. It's also used to boost immunity and prevent and cure bacterial [24] and fungal infections [5]. The plant treats fever, cough, headache, abdominal pain, sinus congestion, gout, rheumatism, hemorrhoids, asthma, bronchitis, shortness of breath, low blood pressure, low blood sugar, high blood sugar, and snake bites [2], as well as maintaining healthy liver function, asthma, arthritis, back pain, bronchitis, chronic fever, tuberculosis, rhinitis, malaria, and stubborn skin conditions such as leprosy [25]. Garlic cloves have antilipemic (cholesterol-lowering), antihypertensive, antimicrobial, and anticancer qualities, all of which help prevent cancer cells from forming in the stomach, liver, and other human organs [2], and also help with asthma, arthritis, back pain, bronchitis, chronic fever, tuberculosis, rhinitis, malaria, stubborn skin conditions including leprosy, leucoderma, skin discoloration, and itching colic, enlarged spleen, hemorrhoids, fistula, bone fracture, gout, urinary tract disease, diabetes, and kidney stone [9]. The plant also has antibacterial and anthelmintic capabilities, as well as diuretic, digestive, and vaginal infections, platelet effects, sickle cell anemia, liver-protecting, and detoxifying properties, as well as antioxidant and radiation-protecting properties [1].

### 3.4. Antidiabetic Activity

In diabetic mice, garlic extract dramatically reduced serum glucose, total cholesterol, triglycerides, urea, uric acid, aspartate aminotransferase, and alanine aminotransferase while significantly increasing serum insulin [26]. It also works to lower serum glucose levels in diabetic rabbits, rats, and mice that have been induced with STZ or alloxan [27]. The volatile sulfur components in garlic are primarily responsible for its antidiabetic action. Garlic has also been demonstrated to help with insulin resistance management [28].

### 3.5. Antioxidant Activity

The presence of diallyl sulfide (DAS) and diallyl disulfide (DADS), as well as s-ethylcysteine (SEC) and n-acetylcysteine (NAC), protected against lipid-related oxidation by activating antioxidant enzymes. Garlic is high in antioxidants, which aid in the destruction of free radical particles that damage cell membranes and DNA, contributing to the aging process. Garlic is high in antioxidants, which assist to fight free radicals, which can damage cell membranes and DNA and speed up the aging process [29, 30].

### 3.6. Hepatoprotective Activity

Garlic is an antioxidant that can be used to treat alcoholic diseases, although it is an inefficient treatment. Marker enzymes for liver function and integrity include ALT, AST, and ALP. Lead administration resulted in a considerable rise in plasma ALT and ALP activities, as well as a reduction in plasma AST activity. The activity of ALT and ALP was greatly lowered after draining garlic, while the activity of AST was significantly elevated [30]. In rats, aged garlic and garlic diallyl sulfur compounds protected them from hepatotoxicity caused by chemicals. Aflatoxin B1 mutagenesis effects are avoided by aged-garlic extracts, which have been proven to reduce both the production and bioactivation of liver carcinogenic nitrosamines [9].

### 3.7. Anti-Inflammatory Activity

The anti-inflammatory properties were attributed to a reduction in the expression and production of the proinflammatory cytokines TNF- and IL-1 [31]. The presence of bioactive components such as diallyl sulfide, which is a proinflammatory cytokine that inhibits TNF and IL-1 secretion, and allyl methyl sulfide, which has been shown to stimulate anti-inflammatory cytokine and IL-10 production, could explain the regulation of proinflammatory and anti-inflammatory cytokine levels in the colon through garlic oil treatment [32].

### 3.8. Cardiovascular Activity

Garlic consumption lowers blood pressure, inhibits atherosclerosis, decreases serum cholesterol and triglycerides, suppresses platelet aggregation, and increases fibrinolytic activity, among other things [10]. Garlic also inhibits the formation of 3-hydroxy-3-methylglutaryl-CoA, which lowers cholesterol levels. Garlic has been found to decrease LDL oxidation, platelet aggregation, and arterial plaque development, as well as lower homocysteine, lower blood pressure, and improve microcirculation, all of which are crucial in diabetes. Garlic can also aid in preventing cognitive decline by shielding neurons from neurotoxicity and apoptosis, which helps to prevent ischemia, obsessive-compulsive disorder (OCD), and neuronal death while also boosting learning and memory retention [33].

### 3.9. Hyperlipidemia

Garlic inhibited liver lipogenesis and cholesterol-forming enzymes like malic acid synthetase, fatty acid synthetase, glucose-6-phosphate dehydrogenase, and 3-hydroxy-3-methylglutaryl-CoA (HMG-CoA) reductase. Water-soluble organosulfur compounds, such as S-allyl cysteine (SAC) found in mature garlic extract and diallyl disulfide (DADS) found in garlic oil, are effective inhibitors of cholesterol formation, according to in vitro research. To avoid plaque formation, everyone should be able to cut cholesterol and reduce lipid peroxidation. Low-density lipoproteins (LDL) are suppressed and LDL resistance to oxidation is increased, according to in vitro investigations [34].

### 3.10. Antibacterial Activity

Garlic extract inhibits the growth of Gram-positive and Gram-negative bacteria such as *Staphylococcus*, *Streptococcus*, *Micrococcus*, *Enterobacter*, *Escherichia*, *Klebsiella*, *Lactobacillus*, *Pseudomonas*, *Shigella*, *Salmonella*, *Proteus*, and *Helicobacter pylori*. This inhibition of garlic extracts due to the presence of enzymes in allicin activity is produced by alliinase [35].

### 3.11. Antiviral Activity

Garlic and its sulfur components demonstrated antiviral action against *coxsackievirus* spp., herpes simplex virus types 1 and 2, influenza B, parainfluenza virus type 3, *vaccinia* virus, vesicular stomatitis virus, human immunodeficiency virus type 1, and human rhinovirus rype 2. In general, the sequence of a virucidal effect was as follows: ajoene > allicin > allyl methyl thiosulfinate > methyl allyl thiosulfinate > methyl allyl [23].

### 3.12. Antifungal Activity

Both the aqueous garlic extract and the concentrated garlic oil showed an inhibitory effect against *Aspergillus* [36]. Allicin showed a fungicidal effect against numerous yeasts and fungi, including *Candida albicans*, *Cryptococcus trichophyton*, *Histoplasma capsulatum*, and *Cryptococcus neoformans* [37]. Garlic has been shown to inhibit the growth of fungal diseases.

### 3.13. Anticancer Activity

The study looked at the effects of garlic on cell lines from leukemia, melanoma, and neuroblastoma. Garlic's flavor is characterized by allyl sulfides. In experimental carcinogenesis in various malignancies, these drugs suppress both the start and promotion stages of tumorigenesis [38]. People who consumed more garlic had a 54% decreased risk of pancreatic cancer than those who consumed fewer amounts of garlic [39]. Garlic supplements such as fresh garlic extract, matured garlic, garlic oil, and many organosulfur compounds generated from garlic have all been demonstrated to have chemopreventive benefits. The presence of organosulfur compounds in garlic is thought to be responsible for the chemopreventive effect. Free radical scavenging activity was discovered in aged garlic extract but not in fresh garlic extract. S-allyl cysteine and S-allyl mercapto-L-cysteine, the two major components in aged garlic, showed the highest free radical scavenging activity. Furthermore, several organosulfur compounds produced from garlic, such as acetylcysteine, have been shown in numerous animals to slow the growth of chemically created and transplantable cancers [40].

### 3.14. Antihypertensive Activity

Garlic supplements may help hypertensive people reduce their blood pressure and oxidative stress [41]. An in vitro study has proven that garlic sulfur compounds, which are produced when red blood cells convert the organic polysulfide of garlic to hydrogen sulfide, have vasoactive properties and are a recognized endogenous cardiovascular protective vascular cell-signaling molecule [3].

### 3.15. Antiplatelet Effect

Garlic supplements have been demonstrated to reduce cyclooxygenase activity and thromboxane A2 production, resulting in antiplatelet action [42]. Garlic has been shown to inhibit platelet aggregation in both in vitro and in vivo experiments. Garlic's antithrombotic impact is investigated in situ by modifying fibrinolytic activity through enhanced plasminogen activation and inhibition of thrombin production [43].

### 3.16. Anthelmintic Activity

In vitro, an alcoholic extract showed moderate anthelmintic efficacy against human *Ascaris lumbricoides*. Garlic is effective in the treatment of dysentery and serves as a wormery. Garlic oil is also said to have anthelmintic properties, which means it kills all dangerous parasites in the intestines. Garlic can help you get rid of intestinal worms. Garlic's sulfurous components can help you get rid of tapeworms [31]. The allicin component of garlic has antihelmintic properties against *Ascaridia galli* in chickens (the main active ingredient in garlic). *A. galli* and *Heterakis gallinarum* died after being exposed to garlic oil. In both parasites, garlic extract dramatically reduced glucose uptake, glycogen content, and oxygen use [44].

### 3.17. Reduces Stress

In hyperglycemic rats, aged garlic extract prevents adrenal hypertrophy, hyperglycemia, and elevations in corticosterone produced by immobilization stress [8].

### 3.18. Sickle Cell Anemia

Because aged garlic extract (AGE) and other components of AGE, such as S-allylcysteine (SAC) and fructosyl arginine, are known to have antioxidant effects, they can prevent the production of dense cells. However, like many other garlic components, antioxidant activity has been demonstrated. The combination of aged garlic extract (4.0 mg/ml) and other helpful nutrients such as black tea extract, green tea extract, Pycnogenol, lipoic acid, vitamin E, coenzyme Q10, and carotene [45] inhibited dense cell development by 50%. Garlic's unfavorable side effects are that garlic use can cause bad breath, body odor, nausea, vomiting, gas, weight loss, face flushing, tachycardia, disorientation, sleeplessness, and allergic responses. The entire tuber generates very little juice; yet, it is potent and can be used as an emetic in modest doses. Although garlic is generally thought to be safe, there have been cases of topical garlic burns [46].

### 3.19. Vaginal Infections

Garlic is one of the most effective antibiotics. It has bactericidal and fungicidal qualities and can kill or limit the growth of bacteria that cause vaginal discomfort, vaginitis, and vaginal flow [23]. To heal this condition, many garlic cloves were consumed. Scabies can also be treated with it. In women of childbearing age, bacterial vaginosis (BV) is one of the most common causes of vaginal discharge. Garlic can be used as a natural treatment for BV. In vitro, garlic extract reduced the development of Gram-positive and Gram-negative bacteria [23].

## 4. Discussion

Since ancient times, garlic (*Allium sativum* L.) has been associated with a variety of biological processes. Garlic contains many biologically active components that help it to be used in pharmaceuticals. In addition, it includes important minerals, vitamins, and proteins and is well-known as a spice or spice in continental cuisine; it also has a variety of possible pharmacological effects against a variety of life-threatening diseases and disorders, along with this plant [8, 42].

Garlic is used to treat a variety of diseases around the world, including high blood pressure, infections, and snakebites, and it has also been used to ward off evil spirits and has antimicrobial effects in some cultures [47]. Garlic inhibits and destroys bacteria, fungus, and parasites, as well as lowers blood pressure, cholesterol, and sugar levels, preventing blood clotting and protecting the liver. It also has anticancer effects [8]. Garlic can also help to improve the immune system, prevent sickness, and preserve good health. It can activate the lymphatic system, which aids in the evacuation of waste from the body. Furthermore, it is a powerful antioxidant and can help protect cells from free radical damage [25]. In particular, the heart, stomach, circulation, and lungs are also aided and supported by it [23]. Garlic is an efficient natural agent in the treatment of wound infections caused by the common cold, malaria, cough, and pulmonary TB, as well as high blood pressure, sexually transmitted diseases, mental disorders, kidney and liver problems, asthma, and diabetes [6]. Excessive alcohol use reduces oxygen radical production, resulting in a reduction in the body's normal defensive mechanisms, altered enzyme action, decreased DNA repair, decreased oxygen consumption, lipid peroxidation, and protein oxidation. Raw garlic is an antioxidant that protects tissue from oxidative stress when taken orally [48].

## 5. Conclusion

The main biologically active ingredient of garlic (allicin) and its derivatives have significant nutritional and medicinal uses around the world. It's a low-toxicity, safe, and abundant source of physiologically active chemicals. Since ancient times, all portions of the plant have been used as a spice or seasoning for sausages and salads, as well as in folk medicine. The therapeutic benefits of garlic are due to a higher concentration of sulfur compounds in the plant. As a result, more pharmacological research is needed to determine its medical efficacy in improving human health.

## Data Availability

The data generated or analyzed during this study were included in the mother document; therefore, no additional data are available.

## Abbreviations

AGE: Aged garlic extract
ALP: Alkaline phosphatase
ALT: Alanine transaminase
AMS: Allyl methyl sulfide
AST: Aspartate transaminase
BV: Bacterial vaginosis
NAC: N-acetylcysteine
DAS: Diallyl sulfide
DADS: Diallyl disulfide
DNA: Deoxyribonucleic acid
LDL: Low-density lipoproteins
IL-1: Interleukin-1
OCD: Obsessive-compulsive disorder
SEC: S-ethylcysteine
TNF: Tumfactor-alpha factor alpha.
